# Development of a New Risk Score for Incident Type 2 Diabetes Using Updated Diagnostic Criteria in Middle-Aged and Older Chinese

**DOI:** 10.1371/journal.pone.0097042

**Published:** 2014-05-12

**Authors:** Xingwang Ye, Geng Zong, Xin Liu, Gang Liu, Wei Gan, Jingwen Zhu, Ling Lu, Liang Sun, Huaixing Li, Frank B. Hu, Xu Lin

**Affiliations:** 1 Key Laboratory of Nutrition and Metabolism, Institute for Nutritional Sciences, Shanghai Institutes for Biological Sciences, Chinese Academy of Sciences and University of Chinese Academy of Sciences, Shanghai, China; 2 SIBS-Novo Nordisk Translational Research Centre for PreDiabetes, Shanghai Institutes for Biological Sciences, Chinese Academy of Sciences, Shanghai, China; 3 Departments of Nutrition and Epidemiology, Harvard School of Public Health, Boston, Massachusetts, United States of America; Medical University Innsbruck, Austria

## Abstract

Type 2 diabetes mellitus (T2DM) reaches an epidemic proportion among adults in China. However, no simple score has been created for the prediction of T2DM incidence diagnosed by updated criteria with hemoglobin A1c (HbA1c) ≥6.5% included in Chinese. In a 6-year follow-up cohort in Beijing and Shanghai, China, we recruited a total of 2529 adults aged 50–70 years in 2005 and followed them up in 2011. Fasting plasma glucose (FPG), HbA1c, and C-reactive protein (CRP) were measured and incident diabetes was identified by the recently updated criteria. Of the 1912 participants without T2DM at baseline, 924 were identified as having T2DM at follow-up, and most of them (72.4%) were diagnosed using the HbA1c criterion. Baseline body mass index, FPG, HbA1c, CRP, hypertension, and female gender were all significantly associated with incident T2DM. Based upon these risk factors, a simple score was developed with an estimated area under the receiver operating characteristic curve of 0.714 (95% confidence interval: 0.691, 0.737), which performed better than most of existing risk score models developed for eastern Asian populations. This simple, newly constructed score of six parameters may be useful in predicting T2DM in middle-aged and older Chinese.

## Introduction

The type 2 diabetes mellitus (T2DM) epidemic has been documented globally in recent decades, particularly in Asian populations with rapid nutrition and lifestyle transitions [1]. Additionally, Asians have been associated with higher susceptibility to developing T2DM, despite relatively lower adiposity, than Caucasians [2]. Moreover, compared with other Asian groups in a multiethnic study, Chinese were shown to have stronger correlations of body mass index (BMI) and waistline with unfavorable status of insulin resistance, inflammation and adiponectin, which could precede T2DM [3]. Because the onset of T2DM often occurs several years before clinical diagnosis [4]. It remains to be elucidated whether ethnic- or country- specific screening methods are required for early diagnosis of and intervention for T2DM.

In addition to the plasma glucose criteria for T2DM diagnosis, either using fasting plasma glucose (FPG) or the 2-hour value from a 75-g oral glucose tolerance test (OGTT), the American Diabetes Association (ADA) and World Health Organization recently adopted hemoglobin A1c (HbA1c) ≥6.5% as one criterion for diagnosing T2DM [5]. It was reported that the prevalence of diabetes diagnosed by glucose and HbA1c varied substantially in different ethnic populations [6], [7].

Large interventions on individuals at high risk for T2DM demonstrated that lifestyle intervention can reduce or delay development of T2DM [8]. Numerous diabetes risk scores [9], therefore, have been developed for screening high risk individuals for early intervention. However, only a small proportion of scores were developed based on longitudinal cohorts, especially in eastern Asia [9]–[16]. Additionally, the predictive power of a T2DM risk score developed from one population may not apply to other populations [17]. Furthermore, whether non-traditional T2DM risk factors, such as C-reactive protein (CRP) can improve predictive power of such scores still remains controversial [18].

To our knowledge, no risk model based on prospective studies had been constructed for Chinese populations when HbA1c ≥6.5% was adopted for T2DM diagnosis. Therefore, in this study, we aimed to identify baseline risk factors associated with incident T2DM, and develop a simple points-based score to estimate T2DM risk after a 6-year follow-up in middle-aged and older Chinese.

## Methods

The design and recruitment of participants at baseline have been described in detail elsewhere [19]. In brief, a total of 3,289 eligible participants (1,458 men and 1,831 women) aged 50–70 years from local communities in both urban and rural areas in Beijing and Shanghai, China were recruited in 2005. Face-to-face interviews were conducted by trained staff to collect data about demographic variables, health status, medication use, family history of chronic diseases, and health behavior (smoking, alcohol use and physical activity). All participants were required to fast overnight (≥7 h) before having a physical examination. Measurements of body weight and height, waist and hip circumference, and blood pressure have been described previously [19]. Fasting peripheral venous blood samples were collected in tubes containing EDTA as anticoagulant, aliquoted, and stored at -80°C until analysis [19].

In 2011, the 6-year follow-up survey was conducted simultaneously in both Beijing and Shanghai. Participants were considered to be lost to follow-up if they could not be contacted or refused to participate. For individuals unable to fully participate due to illness or other reasons, a shortened questionnaire was used to collect information about the onset of age-related diseases including diabetes, cardiovascular diseases (CVD), and cancers. For individuals who died during the 6-year period, information about causes of mortality including diabetes and its complications was collected. At home, a face-to-face interview was conducted by trained staff using standardized questionnaires similar to that used at baseline. A family member was asked to answer questions if a participant was unable to respond due to cognitive problems or severe illness. Physical examinations and venous blood sample collections after overnight fasting were conducted using the same protocol as that used at baseline. The study was approved by the Institutional Review Board of the Institute for Nutritional Sciences, and a written informed consent was obtained from every participant.

During the follow-up period, 760 (23.1%) participants were considered as lost to follow-up due to either failure to be contacted (n = 554) or reluctance to participate (n = 206). Most characteristics were comparable between participants followed and those lost to follow-up [20]. Of the followed participants (n = 2,529), 84 completed only the shortened questionnaire, and 122 died.

BMI was calculated as weight (kg)/height^2^ (m^2^). Blood pressure was measured three times and the 2nd and 3rd measures were averaged for analysis [19]. Hypertension was defined as “yes” if one had systolic blood pressure ≥140 mmHg, or diastolic blood pressure ≥90 mmHg, or if antihypertensive medication was used.

Baseline FPG, total cholesterol, high-density lipoprotein (HDL) cholesterol, low-density lipoprotein (LDL) cholesterol and triglycerides were measured by commercial reagents (Wako Pure Chemical Industries, Osaka, Japan) on an automatic analyzer (Hitachi 7080, Tokyo, Japan) [19]. HbA1c was quantified from resolved erythrocyte with an automated immunoassay (Tina-quant Hemoglobin A1c II; Roche Diagnostics, Indianapolis, Indiana, USA). The HbA1c assay has been standardized against the approved IFCC reference method and can be transferred to results traceable to DCCT/NGSP. Plasma high sensitive CRP concentration was determined by a particle-enhanced immunoturbidimetric assay (Ultrasensitive CRP kit, Orion Diagnostica, Espoo, Finland) [19]. Reagents from the same companies and identical assay procedures were used for each above mentioned biomarkers at baseline and in the 6-year follow-up.

In the current study, T2DM was diagnosed according to the ADA recommended criteria [5] and classified as follows: (1) FPG ≥7.0 mmol/L alone; (2) HbA1c ≥6.5% alone; (3) FPG ≥7.0 mmol/L and HbA1c ≥6.5%; or (4) self-reported diagnosis of diabetes or use of anti-diabetic medications within the 6-year follow-up period. Individuals were excluded if they satisfied any one of the above criteria at baseline (n = 428), did not report having diabetes, or failed to provide fasting blood in the follow-up survey (n = 189). Therefore, a total of 1,912 participants were included in the current analysis.

### Statistical analyses

Baseline characteristics of included participants without diabetes were compared between men and women, using *Chi*-square tests for categorical variables and *t*-tests for continuous variables. Plasma triglycerides and CRP levels were (natural log) transformed to remove skewness before analyses.

Backward elimination of stepwise logistic regressions was used to investigate significant risk factors for predicting incident T2DM within 6 years. Risk factors suggested by previous literature, including age, sex, lifestyle (smoking, alcohol use and physical activity), hypertension, BMI, glucose metabolism (FPG and HbA1c), blood lipids (triglycerides, HDL cholesterol, and total cholesterol) and CRP were entered in the initial model simultaneously. Only statistically significant risk factors were retained in the final model. The backward elimination was repeated using waist circumference, or waist-to-hip ratio to replace BMI. Areas under the receiver operating characteristic curve (AUC) were used to evaluate the discriminatory capacities of these models.

The discrimination of CRP, a non-traditional risk factor of CVD and diabetes, in predicting T2DM was further investigated by comparing the AUC with or without it in the prediction model. Category-free net reclassification improvement (NRI) and integrated discrimination improvement (IDI) were also calculated to estimate the contribution of CRP to the prediction model [21]. The final model was internally validated by estimation of the AUC using a tenfold cross-validation procedure. The whole sample was randomly split into ten equal parts. For k = 1, … , 10, the kth part was used as the validation dataset and the remaining nine parts as the training dataset. For each partition of the validation and training sets, the coefficients from the training set was obtained and assigned to the validation set to evaluate performance by estimating the AUC for the model. The procedure was repeated for 100 times, and AUC and 95% confidence interval (CI) were averaged to correct over-fit of the model.

A simple point system for estimating T2DM risk within 6 years was then built according to the procedures as described in detail by Sullivan and colleagues [22]. First, clinically meaningful categories and associated medians for each continuous predictor were created and calculated. Second, reference groups for each categorized predictor were separately defined. Third, beta regression coefficients for continuous and categorical predictors were obtained and a constant to reflect the sex difference for T2DM risk was set. Fourth, the point score for each category of predictors was estimated using the product of the corresponding regression coefficients and how far the median of each category was from their reference group. The point range was calculated based on the points for each predictor. Finally, the 6-year risk of T2DM was estimated based on the score and the constant reflecting sex difference for T2DM risk.

The C statistic, which is identical to AUC for binary outcomes such as T2DM, was also calculated for several reported risk models developed in prospective studies in eastern Asians, including Chinese (Sun 2009 [10], Chuang 2011 [12], and Liu 2011[13]), Japanese (Doi 2011 [14], and Heianza 2012 [16]) and Koreans (Lim 2012 [15]). The calibration feature of the prediction models was estimated by Hosmer-Lemeshow test, in which a non-significant *P* value indicates good agreement between observed outcomes and model based predictions. The optimal cut-off point for each model was a value that maximized the sum of sensitivity and specificity. Brier score [23], a global summary statistic, sensitivity, and specificity were also calculated.

A two-sided *P* value<0.05 was considered statistically significant. All statistical analyses were performed with Stata 9.2 SE (StataCorp, College Station, Texas, USA).

## Results

### Baseline characteristics

The baseline clinical characteristics of the participants without T2DM are shown in Table 1. With a mean age of 58.1 years (standard deviation: 6.0) of the studied sample at baseline, approximately half of men and women had hypertension. Compared to men, women had significantly higher levels of BMI, HbA1c, and lipids (triglycerides, HDL-, LDL- and total cholesterol), but lower waist circumference, waist-to-hip ratio and FPG.

**Table 1:** Baseline characteristics of the followed participants without diabetes in the 6-year follow-up cohort in Beijing and Shanghai, China, 2005–2011.

	Men	Women	*P* value
n	802	1110	
Age (years)	58.3 (5.9)	57.9 (6.0)	0.159
Hypertension (n, %)	397 (49.5)	563 (50.7)	0.599
BMI (kg/m^2^)	24.0 (3.3)	24.5 (3.6)	0.002
Waist circumference	84.8 (10.5)	81.5 (10.1)	<0.001
Waist-to-hip ratio	0.914 (0.068)	0.867 (0.070)	<0.001
Fasting plasma glucose (mmol/L)	5.38 (0.56)	5.30 (0.54)	0.003
HbA1c (%)	5.62 (0.36)	5.69 (0.36)	<0.001
Triglycerides (mmol/L)^a^	1.04 (0.99, 1.08)	1.10 (1.07, 1.14)	0.032
HDL cholesterol (mmol/L)	1.24 (0.34)	1.34 (0.32)	<0.001
LDL cholesterol (mmol/L)	3.00 (0.89)	3.32 (0.93)	<0.001
Total cholesterol (mmol/L)	4.42 (0.90)	4.76 (0.93)	<0.001
C-reactive protein (mmol/L)^a^	0.64 (0.59, 0.69)	0.62 (0.58, 067)	0.636

Abbreviations: BMI, body mass index; HDL, high-density lipoprotein; LDL, low-density lipoprotein.

Data are adjusted means (standard deviation) unless specified. ^a^Values are geometric means (95% confidence interval). Chi-square tests were used to compare categorical variables and t-tests were used to compare continuous variables.

### T2DM incident rates

Approximately half of the participants (48.3%) without T2DM at baseline were identified to have T2DM in the 6-year follow-up. Among participants with incident diabetes (n = 924), approximately 19.0% met only the FPG ≥7.0 mmol/L criterion (n = 176), 47.6% met only the HbA1c% ≥6.5 criterion (n = 440), 24.8% fulfilled both criteria (n = 229), and the rest (n = 79) were self-reported to have diabetes or had used anti-diabetic medications during the follow-up.

### Baseline risk factors predicting incident T2DM

By utilizing backward elimination of stepwise logistic regressions, we investigated baseline risk factors for predicting incident T2DM within 6 years. Female gender, hypertension, greater BMI, FPG, HbA1c, and CRP were all significantly associated with higher future risk for T2DM, whereas age, lifestyle (smoking, alcohol use and physical activity), and blood lipids (triglycerides, HDL cholesterol, and total cholesterol) were not significant risk factors (data not shown). The same pattern of risk factors was identified when waist circumference, or waist-to-hip ratio, rather than BMI, was included in the model.

### AUC, validation, NRI and IDI

The naïve AUC was 0.728 (95% confidence interval [CI]: 0.705, 0.751) for the logistic model with the significant risk factors including sex, hypertension, BMI, FPG, HbA1c, and log-transformed CRP as predicting variables. The AUC was not significantly reduced when CRP was removed from the model (*P* = 0.5640). Nevertheless, CRP significantly improved the integrated discrimination (*P* = 0.0160) and categorical-free NRI (*P* = 0.0051). Therefore, CRP was kept in for the development of diabetic risk score. The tenfold cross-validated AUC was 0.723 (95% CI: 0.701, 0.746), which was quite similar to the naïve AUC, suggesting that the logistic model was not over-fitted. Neither waist circumference nor waist-to-hip ratio further improved the AUC, NRI and IDI when BMI was already included in the model (all P values>0.05, data not shown).

### Development of risk scores for the prediction of T2DM within 6 years

A simple point system was then created based on the logistic regression coefficients and reference values of each significant risk factor (Table 2). The scores could be manually counted to estimate the risk for future T2DM within 6 years. In the current study, 28.5% had a risk below 35%, 32% had a risk between 35% and 50%, and 39.5% had a risk of 50% or higher using this score system.

**Table 2:** Algorithm to estimate risk for incident type 2 diabetes mellitus using total points for the categorical model with logistic regression analysis in the 6-year follow-up cohort in Beijing and Shanghai, China, 2005-2011.

Items	Reference value (*W_ij_*)	*β_i_*	*P value*	*β_i_(W_ij_-W_iREF_)*	Point*_ij_* = *β_i_(W_ij_-W_iREF_)/B* a
Sex		0.2271 ( = *B*)	0.027		
Male	0 (*W_1REF_*)			0	0
Female	1			0.2271	1
Body mass index (kg/m^2^)		0.0533	0.001		
<24	21.4 (*W_2REF_*_)_			0	0
24 to <28	25.8			0.2345	1
≥28	29.2			0.4157	2
Fasting plasma glucose (mmol/L)		0.9624	<0.001		
<5.6	5.1 (*W_3REF_*_)_			0	0
5.6 to <6.1	5.8			0.6437	3
­ ≥ 6.1	6.4			1.2511	6
HbA1c (%)		1.3375	<0.001		
<5.5	5.3 (*W_4REF_*_)_			0	0
5.5 to <5.9	5.7			0.5350	2
≥5.9	6.1			1.0700	5
Hypertension (%)		0.2420	0.019		
No	0 (*W_5REF_*_)_			0	0
Yes	1			0.2420	1
C-reactive protein (mmol/L)^b^		0.1104	0.019		
<0.39	-2.12^c^			0	0
0.39 to 0.99	-0.49			0.1800	1
≥1.00	0.65			0.3058	2
Maxim total point					17
**Total point**	0 1 2 3 4 5 6 7 8 9 10 11 12 13 14 15 16 17				
**6-year risk of type 2 diabetes, %**	0.20 0.24 0.29 0.34 0.39 0.44 0.50 0.56 0.61 0.66 0.71 0.76 0.80 0.83 0.86 0.89 0.91 0.92				

a The points are rounded to the nearest integer.

b C-reactive protein was tertiled in the current sample.

c The reference figure was the median of natural log transformed values in each C-reactive protein category.

### Comparisons with other prediction models developed for eastern Asians

**Figure 1:**
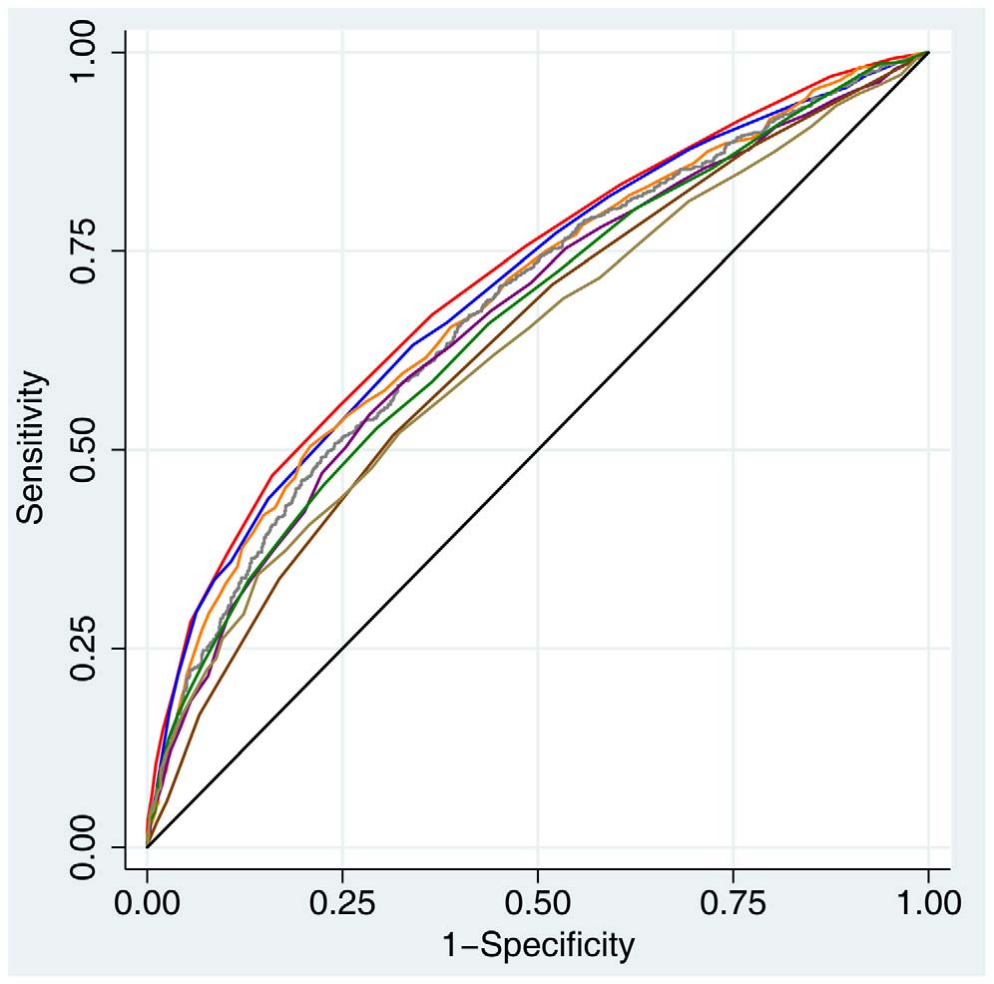
Receiver Operating Characteristic Curves for Various Models Applied to the Study Population in the 6-Year Follow-Up Cohort in Beijing and Shanghai, China, 2005–2011. Red, this study (area under the curve [AUC]: 0.714); Blue, Heianza (AUC: 0.701); Orange, Lim (AUC: 0.689); Gray, Sun (AUC: 0.678); Purple, Chien (AUC: 0.665); Green, Chuang (AUC: 0.661); Pink, Liu (AUC: 0.634); Brown, Doi (AUC: 0.631); Black, reference (AUC: 0.500).

The risk factors included in prediction models from Asian samples are shown in Table S1. The AUC based on the simple point model (0.714 [95% CI: 0.691, 0.737]) tended to be higher than that based on the model developed in a Japanese study by Heianza et al (AUC: 0.701 [95% CI 0.678, 0.924], *P* = 0.086) and was significantly higher (all *P*<0.05) than others from models developed in eastern Asian populations including Chinese (Figure 1). As shown in Table 3, the current simple point model also had the lowest Brier score, indicating a good overall discriminatory ability. With an optimal cut-off value of 7, the current simple point model had the highest Youden index, the highest proportion of correctly classified participants, and highest positive likelihood ratios among all the models.

**Table 3:** C statistic, sensitivity, specificity, Youden index and likelihood ratios of the optimal cut-off value for the risk of diabetes in each model for Eastern Asians in the 6-year follow-up cohort in Beijing and Shanghai, China, 2005–2011.

Model	C statistic	Hosmer–Lemeshow p value	Brier score	Optimal cut-off value	Sensitivity	Specificity	Youden index	Correctly classified	LR+	LR-
Sun et al., 2009	0.678^a^	0.1910	0.226	≥15.6	0.509	0.759	0.268	0.638	2.11	0.65
Chien et al., 2009	0.664^a^	0.4304	0.229	≥6	0.547	0.712	0.258	0.632	1.89	0.64
Chuang et al., 2011	0.661^a^	0.8500	0.229	≥12	0.527	0.707	0.234	0.620	1.80	0.67
Liu et al., 2011	0.634^a^	0.6021	0.236	≥3	0.518	0.685	0.204	0.605	1.65	0.70
Doi et al., 2011	0.631^a^	0.1921	0.235	≥19	0.343	0.858	0.201	0.609	2.42	0.77
Lim et al., 2012	0.689^a^	0.0900	0.222	≥33	0.504	0.792	0.296	0.653	2.42	0.63
Heianza et al., 2012	0.701	0.0255	0.218	≥11	0.632	0.660	0.292	0.646	1.86	0.56
Current study	0.714	0.6166	0.213	≥7	0.555	0.754	0.309	0.658	2.26	0.59

Abbreviations: LR+, likelihood ratio for a positive test results; LR-, likelihood ratio for a negative test results.

Higher discrimination C statistics was well as lower Brier scores indicated better performance.

a C-statistic was significantly lower (P<0.05) when compared to the current study.

## Discussion

In this population-based prospective study with middle-aged and older Chinese, a high rate of incident T2DM was observed after a 6-year follow-up. Among the incident T2DM cases, nearly half of them were identified using only the HbA1c criterion. Female gender, hypertension, BMI, FPG, HbA1c, and CRP were all significantly associated with incident T2DM during the 6-year follow-up period. A simple score based on these significant risk factors performed better in predicting risk for 6-year T2DM than most of the models developed in eastern Asia populations.

### Diagnosis of T2DM by adopting HbA1c criterion

HbA1c is traditionally used to monitor glycemic control in diabetic patients. Because the HbA1c assay has been improved and standardized, an International Expert Committee recommended HbA1c as one criterion for diagnosing diabetes in 2009 [24]. Subsequently, this suggestion was adopted by the ADA in 2010 and the World Health Organization in 2011 [5]. However, whether HbA1c ≥6.5% should be used as a cut-off point for diagnosing diabetes still remains controversial, because some studies suggest that HbA1c levels can vary among different ethnic/racial groups [25], [26]. Nevertheless, a recent analysis of the data from the 2005–2008 National Health and Nutrition Examination Survey did not support the use of ethnic-specific HbA_1c_ cut-off points for diagnosing diabetes mellitus [27]. Findings from a prospective study in Japan also supported the use of HbA1c ≥6.5% criterion [28].

It was noteworthy in our study that a larger proportion of incident diabetes was diagnosed exclusively by HbA1c ≥6.5% rather than by FPG. Consistent with our results, in a nationally representative sample of 98,658 Chinese adults in 2010, the HbA1c identified the highest proportion of diabetes relative to FPG and OGTT in middle-aged and older groups (50–59, 60–69, and ≥70 years) [29]. In a study of 35,624 non-diabetic Koreans aged 20–89 years, using HbA1c, FPG or HbA1c+FPG criteria discovered 23.5%, 31.6% and 44.9% of new diabetic cases (n = 1,491), respectively [30]. A similar pattern was also observed in a large study with 26,884 participants aged 20–91 years from Japan [31]. The proportion of new diabetic cases (n = 978) diagnosed by FPG, HbA1c, or both were 26.0%, 26.5% and 47.5%, respectively. Taken together, although the proportion of diabetes identified by HbA1c and glucose criteria (FPG or OGTT) may vary in different studies due to differences in study designs and characteristics like age [32], the related evidence suggests that adding HbA1c to the diagnosis criteria could screen a significant proportion of diabetic individuals; whereas using glucose criteria alone is likely inadequate to discover new cases in Chinese and other ethnic populations [29].

### Inflammation and risk for T2DM

Chronic low-graded inflammation, as indicated by CRP levels, has been linked to diabetic risk _ENREF_16[33]. It was noticed that circulating CRP levels are generally lower among Asians than Caucasians and Hispanic populations [19]. Increased CRP, however, was observed to be significantly associated with higher risk for T2DM development in Japanese [34], [35]. Previous cross-sectional studies have linked CRP with prediabetes [36] and diabetes [37] in Chinese. To our knowledge, no prospective data in this regard have been collected in Chinese population so far. In line with the studies of Japanese and other populations, the current study showed that CRP was independently associated with future T2DM risk even after adjustment for baseline BMI, FPG, and HbA1c.

Few studies have estimated to what extent CRP could improve the discrimination power of models containing conventional risk factors for diabetes [18]. Interestingly, our study demonstrated that adding CRP enhanced capabilities of reclassification and discrimination for the diabetic prediction model. Salomaa et al. reported that CRP improved IDI in the FINRISK97 cohort, but did not improve IDI in the Health 2000 cohort in Finland [38]. In the Framingham Heart Study, CRP was not observed to improve the T2DM prediction capability after adjustment for several clinical covariates [18]. It remains unknown the factors attributing to the inconsistent findings. Additional studies are needed to validate whether CRP is able to improve prediction of T2DM development, especially in Asians with relatively low circulating CRP levels.

### Risk score for T2DM

The risk score model developed in our study population performed the best among existing models developed in prospective cohorts from eastern Asians, except the one developed in Japanese by Heianza et al. [16]. In their model, Heianza et al included FPG and HbA1c in addition to family history of diabetes, smoking, and BMI to generate the risk score [16]. They also diagnosed T2DM by using HbA1c and FPG criteria [16]. On the other hand, other compared risk models, including the models developed in other Chinese populations, were built on FPG and/or 2-h OGTT based T2DM diagnostic criteria [10]–[15], which may partially explain the low prediction power of those models, since they lacked a sizable proportion of T2DM cases detected by HbA1c alone. Additionally, none of these eastern Asian models has included CRP as a predictive factor.

The risk score could be used to identify new T2DM cases in large-scale epidemiologic studies or in a single clinical visit after fasting overnight. According to a recent national survey in China, it is estimated that nearly 70% of diabetes were previously undiagnosed [39]. Therefore, a simple risk score like the current one would help not only to identify individuals with high risk for future T2DM, but also to screen new T2DM patients because this score requires tests for fasting glucose and HbA1c.

### Strengths and limitations

To our knowledge, this is the first prospective study in China to develop a risk score for T2DM when the updated criteria for T2DM diagnosis were used in Chinese. The components of the simple score model could be obtained from one clinical visit or an epidemiological survey with an overnight fast. The sample size of our study was relatively large with 6-year follow-up duration. The community-based design also reduced biases compared to hospital-based surveys in which non-diabetic patients are often recruited as controls. Nevertheless, several limitations should be mentioned. First, external validation was not performed due to lack of data from studies with similar design in China. Second, because participants were aged 50–70 years at baseline, the simple score may not be generalized to other age groups in Chinese. Third, having only one FPG measurement may increase false positive rate of T2DM. Fourth, because 2-h OGTT is time-consuming and very costly in large epidemiological studies, our study did not measure 2-h OGTT values, which may have caused us to miss a fraction of undiagnosed T2DM. However, according to a recent national survey conducted in China, approximately 85% of newly diagnosed diabetes could be identified by combining FPG and HbA1c criteria [29]. Finally, the HbA1c levels could also be affected by unmeasured factors like abnormal red blood cell turnover rate rather than glucose status. It remains to be elucidated to what degree unmeasured factors could influence the incident rate of T2DM among middle-aged and older adults.

### Conclusions

In this population-based prospective study in China, a large proportion of incident T2DM could be identified using the cut-off point of HbA1c ≥6.5% after a 6-year follow-up. A simple score constructed based on FPG, HbA1c, CRP as well as sex, BMI and hypertension could be used to screen and to estimate the risk for future T2DM in middle aged and older Chinese.

## Supporting Information

Table S1 **Risk factors included in prediction models.**
(DOC)
