# Correction: Development of a New Risk Score for Incident Type 2 Diabetes Using Updated Diagnostic Criteria in Middle-Aged and Older Chinese

**DOI:** 10.1371/journal.pone.0115929

**Published:** 2014-12-12

**Authors:** The PLOS ONE Staff

There are errors in Table 1 and Table 2. In both tables the unit of C-reactive protein should be mg/L, rather than mmol/L. Please see the corrected Table 1 and Table 2 here.

**Table 1:** Baseline characteristics of the followed participants without diabetes in the 6-year follow-up cohort in Beijing and Shanghai, China, 2005–2011.

	Men	Women	*P* value
n	802	1110	
Age (years)	58.3 (5.9)	57.9 (6.0)	0.159
Hypertension (n, %)	397 (49.5)	563 (50.7)	0.599
BMI (kg/m^2^)	24.0 (3.3)	24.5 (3.6)	0.002
Waist circumference	84.8 (10.5)	81.5 (10.1)	<0.001
Waist-to-hip ratio	0.914 (0.068)	0.867 (0.070)	<0.001
Fasting plasma glucose (mmol/L)	5.38 (0.56)	5.30 (0.54)	0.003
HbA1c (%)	5.62 (0.36)	5.69 (0.36)	<0.001
Triglycerides (mmol/L)^a^	1.04 (0.99, 1.08)	1.10 (1.07, 1.14)	0.032
HDL cholesterol (mmol/L)	1.24 (0.34)	1.34 (0.32)	<0.001
LDL cholesterol (mmol/L)	3.00 (0.89)	3.32 (0.93)	<0.001
Total cholesterol (mmol/L)	4.42 (0.90)	4.76 (0.93)	<0.001
C-reactive protein (mg/L)^a^	0.64 (0.59, 0.69)	0.62 (0.58, 067)	0.636

Abbreviations: BMI, body mass index; HDL, high-density lipoprotein; LDL, low-density lipoprotein.

Data are adjusted means (standard deviation) unless specified. ^a^ Values are geometric means (95% confidence interval). Chi-square tests were used to compare categorical variables and t-tests were used to compare continuous variables.

**Table 2:** Algorithm to estimate risk for incident type 2 diabetes mellitus using total points for the categorical model with logistic regression analysis in the 6-year follow-up cohort in Beijing and Shanghai, China, 2005-2011.

Items	Reference value (*W_ij_*)	*β_i_*	*P value*	*β_i_(W_ij_-W_iREF_)*	Point*_ij_* = *β_i_(W_ij_-W_iREF_)/B* a
Sex		0.2271 ( = *B*)	0.027		
Male	0 (*W_1REF_*)			0	0
Female	1			0.2271	1
Body mass index (kg/m^2^)		0.0533	0.001		
<24	21.4 (*W_2REF_*_)_			0	0
24 to <28	25.8			0.2345	1
≥28	29.2			0.4157	2
Fasting plasma glucose (mmol/L)		0.9624	<0.001		
<5.6	5.1 (*W_3REF_*_)_			0	0
5.6 to <6.1	5.8			0.6437	3
≥ 6.1	6.4			1.2511	6
HbA1c (%)		1.3375	<0.001		
<5.5	5.3 (*W_4REF_*_)_			0	0
5.5 to <5.9	5.7			0.5350	2
≥5.9	6.1			1.0700	5
Hypertension (%)		0.2420	0.019		
No	0 (*W_5REF_*_)_			0	0
Yes	1			0.2420	1
C-reactive protein (mg/L)^b^		0.1104	0.019		
<0.39	-2.12^c^			0	0
0.39 to 0.99	-0.49			0.1800	1
≥1.00	0.65			0.3058	2
Maxim total point					17
**Total point**	0	1	2	3	4	5	6	7	8	9	10	11	12	13	14	15	16	17
**6-year risk of type 2 diabetes, %**	0.20	0.24	0.29	0.34	0.39	0.44	0.50	0.56	0.61	0.66	0.71	0.76	0.80	0.83	0.86	0.89	0.91	0.92

a The points are rounded to the nearest integer.

b C-reactive protein was tertiled in the current sample.

c The reference figure was the median of natural log transformed values in each C-reactive protein category.

## References

[1] Ye X, Zong G, Liu X, Liu G, Gan W, et al (2014) Development of a New Risk Score for Incident Type 2 Diabetes Using Updated Diagnostic Criteria in Middle-Aged and Older Chinese. PLoS ONE 9(5): e97042 doi:10.1371/journal.pone.0097042 24819157 10.1371/journal.pone.0097042 PMC4018395

