# Supplementary material for: Development of a New Risk Score for Incident Type 2 Diabetes Using Updated Diagnostic Criteria in Middle-Aged and Older Chinese
Source: PLoS One. 2014 May 12;9(5):e97042. doi: 10.1371/journal.pone.0097042 (PMC4018395; doi:10.1371/journal.pone.0097042)
Supplement: Table S1 — Risk factors included in prediction models. (DOC) [file pone.0097042.s001.doc]

**Table S1. Risk Factors Included i**n Prediction Models

| **Model** | **Diagnosis of T2DM** | **Risk factors included in the model** |
| --- | --- | --- |
| Sun et al., 2009 | FPG ≥7.0 mmol⁄l or the use of anti-diabetic medications | Sex, education level, age, current smoking status, BMI, waist circumference, family history of type 2 diabetes, hypertension and FPG |
| Chien et al., 2009 | FPG ≥7.0 mmol⁄l or the use of anti-diabetic medications | Age, BMI, triglycerides, HDL-cholesterol, FPG and white blood cell count^a^ |
| Chuang et al., 2011 | FPG ≥7.0 mmol⁄l or the use of anti-diabetic medications | Age, education level, alcohol use, family history of type 2 diabetes, BMI, waist circumference, hypertension, FPG and triglycerides |
| Doi et al., 2011 | FPG ≥7.0 mmol⁄l, 2-h OGTT ≥11.1 mmol⁄l or the use of anti-diabetic medications | Age, sex, family history of type 2 diabetes, waist circumference, BMI, hypertension, current smoking status, physical activity level and FPG |
| Lim et al., 2012 | FPG ≥7.0 mmol⁄l, 2-h OGTT ≥11.1 mmol⁄l, HbA1c ≥6.5% or the use of anti-diabetic medications | Age, current smoking status, BMI, family history of type 2 diabetes, hypertension, FPG, HDL-cholesterol, triglycerides and HbA1c |
| Heianza et al., 2012 | FPG ≥7.0 mmol⁄l, HbA1c ≥6.5% or self-reported diagnosis by clinicians | Current smoking status, BMI, family history of type 2 diabetes, FPG and HbA1c |
| Current study | FPG ≥ 7.0 mmol⁄l, and⁄or HbA1c ≥6.5% and⁄or the use of anti-diabetic medications and/or self-reported diagnosis by clinicians | Sex, BMI, hypertension, FPG, HbA1c and CRP |

Abbreviations: BMI, body mass index; CRP, C-reactive protein; FPG, fasting plasma glucose; HDL, high-density lipoprotein; OGTT, oral glucose tolerance test.

^a^ White blood cell count was not included in the AUC calculation because this variable was not available in the current study.
